# A Study of the Health-Related Quality of Life and Work-Related Stress of White-Collar Migrant Workers 

**DOI:** 10.3390/ijerph9103740

**Published:** 2012-10-19

**Authors:** Su-Ying Tsai

**Affiliations:** Department of Health Management, I-Shou University, Kaohsiung, No. 8, Yida Road., Yanchao Township, Kaohsiung Country 824, Taiwan; Email: sytsai@isu.edu.tw; Tel.: +886-7-615-1100 (ext. 7414); Fax: +886-7-615-5150

**Keywords:** white-collar worker, work-related stress, health-related quality of life, migrant worker

## Abstract

Little is known about the health-related quality of life (HRQoL) and work-related stress and its risk factors among white-collar businessmen and management workers that migrate to high-income developing countries. A structural questionnaire survey was administered to 156 white-collar Taiwanese management personnel of representative companies of their industries in Taiwan, who were assigned long-term job positions in China. Questionnaire content included demographics and medical history, self-reported physical and mental conditions, personal lifestyle and behavior, Beck Depression Inventory, and information on HRQoL. White-collar migrant workers reported a high prevalence of alcohol consumption (72.4%) and perceived work-related stress (62.2%), and a lower prevalence of regular exercise (12.2%). Workers with higher levels of perceived work-related stress reported more alcohol consumption, a history of hyperlipidemia, and a higher prevalence of self-reported neck pain, poor sleep, and mild/moderate/severe depression. In our primary multivariate risk model to determine lifestyle and work-related stress variables and HRQoL, perceived work-related stress and a feeling of depression negatively impacted both the Physical Component Summary (PCS) and Mental Component Summary (MCS) scores of the SF-36 health survey. Hyperlipidemia and self-reported neck pain were associated with significantly lower PCS scores, whereas cardiovascular disease, gastric ulcer, and poor sleep were associated with statistically lower MCS scores. White-collar migrant workers are generally younger with high socioeconomic status. Perceived work-related stress and a feeling of depression indirectly affect HRQoL. Hyperlipidemia, self-reported neck pain, cardiovascular disease, gastric ulcer, and poor sleep also had a significant negative impact on HRQoL.

## 1. Introduction

Worker migration and labor mobility are increasingly common phenomena in many regions of the World. Migrant workers contribute to the economic growth of high-income countries, often serving as the labor force for the dangerous, dirty, and degrading jobs (the “3 Ds”) that national workers are reluctant to perform [1]. Migrant workers are at high risk for hazardous occupational exposures, injuries, and death. Previous studies reported that migrant workers often experience social exclusion, lack of health and safety training, fear of reprisals for demanding better working conditions, linguistic and cultural barriers that minimize the effectiveness of training, incomplete surveillance of foreign workers, and difficulty accessing care and compensation when injured [2]. These factors paint a worrisome picture regarding the health of immigrant workers, and migrant status can be an important source of occupational health inequality. Increasing evidence supports the hypothesis that immigrant workers are the weakest link of the labor market in rich countries, which is especially hazardous in a period of economic crisis such as the current one [1,2,3]. Further, the employment conditions and associated work environment of most blue-collar migrant workers are dangerous. 

Increased stress from competition and the opportunity to gain an international market share have led to the trend for white-collar businessmen or management workers to migrate to high-income developing countries for work. The term ‘white-collar migrant worker’ refers to a salaried professional or an educated worker who performs semi-professional office, administrative, and sales coordination tasks. Little is known, however, about the health-related quality of life (HRQoL), work-related stress, and risk factors of migration processes of white-collar migrant workers. In the present study, we examined a group of Taiwanese white-collar migrant workers from two representative companies in Taiwan that work in mainland China, a relatively homogeneous management personnel population with regard to socioeconomic background. We focused on HRQoL because HRQoL reflects a person’s perception of the quality of their physical and mental health over time and is closely related to the perceived burden of a person’s self-report of chronic disease and behavioral risk factors. From a broader perspective, HRQoL is considered a broad measure of health status and the patient’s point of view is recognized to be an important component for self-assessment of health care outcomes and self-assessed health status, and is a more powerful predictor of mortality and morbidity than many objective measures of health [4]. This study focused on exploring the association between perceived work-related stress, personal lifestyle behaviors, symptoms of depression, sleep status, and HRQoL, because these lifestyles or behaviors are changeable and HRQoL reflects a person’s perception of their health. Moreover, neck and upper extremity pain is reported with high frequency in repetitive work. But psychological factors have received considerable attention during the past decade. In this study, we examined that the association between self-reported pain symptoms and work-related stress and postulated that self-reported pain symptoms would be independently predictive of HRQoL. The findings of the present study will contribute to a better understanding by health personnel of the health status profile of white-collar migrant workers and allow for the development of stress-prevention programs for these workers.

## 2. Methods

### 2.1. Selection of Subjects

A cross-sectional survey of health status and HRQoL among Taiwanese white-collar migrant workers assigned to work in China was conducted from 1 August 2006 to 30 April 2008. All white-collar management migrant workers in China of two Taiwanese enterprises, Company A (computer hardware and electronics manufacturer) and Company F (plastics manufacturer) were invited to participate in this study. 

Company A is a multinational computer hardware and electronics company headquartered in Taiwan. Its products include motherboards, desktops, laptops, monitors, tablet PCs, servers, and mobile phones. It appears in BusinessWeek’s “InfoTech 100”and “Asia’s Top 10 IT Companies” rankings. Company A has manufacturing facilities in Taiwan (Taipei, Lujhu, Nangan, Guishan), China (Suzhou), Mexico (Ciudad Juárez), and the Czech Republic (Ostrava). Company A’s Hi-Tech Park, located in Suzhou, China, covers 540,000 square meters. In this study, we investigated 41 white-collar management migrant workers in the China (Suzhou) manufacturing facility. 

Company F’s main business area is intermediate raw materials for plastics. Company F’s overseas expansion has focused primarily on the United States and China, and it is one of the largest manufacturers of polyvinyl chloride resins in the world. In China, Company F’s manufacturing facility is mostly located in Kunshan, Nantong, Ningbo, and Fujian Province. In this study, we investigated 118 white-collar management migrant workers in Kunshan, Nantong, Ningbo, and Fujian Province, China. We defined white-collar management migrant workers as those with a superior educational level and high compensation level; hence, they were externally assigned to deal with overseas management and provide professional supervision, and they served in their jobs for at least three months. They usually worked about 8 hours a day, and their positions included specific job responsibilities, such that their bonuses were largely tied to individual performance. Two research companies were selected for the study setting upon consideration of company size, stability, and routine external assignment system. Among the eligible 201 white-collar management migrant workers that we contacted during the study period, 156 provided written informed consent and answered self-administered questionnaires (response rate 77.6%). This study was approved by the Institutional Review Board of the E-Da Hospital (Taiwan).

### 2.2. Assessment Instruments and Definitions

An invitation letter introducing the survey regarding health status and HRQoL among white-collar migrant workers was first mailed to Taiwanese factory directors of the China manufacturing facility, and then telephone and face-to face contact was made approximately 1 week later to confirm approval. The factory’s occupational and environmental health staff helped distribute and collect the self-reported questionnaires. Questionnaires were used to collect data on white-collar management migrant workers’ demographics and medical history, self-reported physical and mental conditions, personal lifestyle and behavior, Beck Depression Inventory (BDI), and HRQoL. Mean time required to complete the survey was 15 min. 

#### 2.2.1. Demographics

Participants’ self-reported demographic data were assessed. Level of education was used as a proxy measure for social class and categorized as follows: (1) high school or below or (2) college or above. Marital status was categorized as single, married and living with their spouse (defined as having a spouse that could provide emotional support), married and living without their spouse (defined as having a spouse that could provide long-distance support) and not currently married (including divorced/separated, widowed). Monthly income was treated as individual work salary from this job and was categorized into one of three grades: $1,600–2,000 dollars, $2,001–2,400 dollars, and more than $2,401 dollars per month. 

#### 2.2.2. Personal Lifestyle and Behavior and Perceived Work-Related Stress

Cigarette smoking history, alcohol intake, and exercise habit were used as lifestyle indices. The participants were asked questions about their cigarette smoking history (current-smoker, ex-smoker, and never-smoker); alcohol intake was limited to wine and hard alcohol and was categorized as no habit of alcohol consumption (or frequency of alcohol consumption was only once a week) and a habit of alcohol consumption (frequency of alcohol consumption was more than once a week). Regular exercise was initiated or/and maintained during the month prior to the investigation. Participants were asked, “Have you participated in regular exercise equivalent to a 1-hour walk at least once a week (yes/no) or have you exercised at least 3 times at least once a week to the extent that you breathe deeply or sweat (yes/no)”. 

To assess perceived work-related stress, we adopted concepts discussed in previous studies [5,6,7,8,9]. Hence, we defined work-related stress as that work demands that are not matched to the knowledge, skills, or abilities of the worker, and that challenge their ability to cope. These demands may be related to time pressure or the amount of work (quantitative demands), or may refer to the difficulty of the work (cognitive demands) or the empathy required (emotional demands), or even to the inability to show one’s emotions at work. Participants responded to the following question: “Did you ever experience work-related stress originating from time pressure or the amount of work, or difficulty of the work, or the empathy required in the last 3 months?”

#### 2.2.3. Medical History and Self-Reported Physical and Mental Conditions

Personal medical history was investigated based on completion of a checklist. The participants were asked whether they had been diagnosed by a physician as having a chronic disease (including hypertension, diabetes, cardiovascular disease, hyperlipidemia, metabolic arthritis, and gastric ulcer). Sleep problems were surveyed and were defined as difficulty falling asleep; difficulty remaining asleep (more than twice a week); the belief that one is not getting enough sleep, resulting in a disturbance of daily activities or normal social activities; and the use of medication for insomnia, focusing on symptoms during the preceding 4 weeks. Data of self-reported physical symptoms were collected in the study and included neck pain, low back pain, and headache, focusing on symptoms during the preceding 4 weeks.

#### 2.2.4. Beck Depression Inventory

The BDI was originally designed to measure the depth or intensity of depression in psychiatric patients [10,11]. It has subsequently been used as a community-screening instrument and for clinical research [12]. The BDI-II was a 1996 revision of the BDI [13], developed in response to the American Psychiatric Association’s publication of the Diagnostic and Statistical Manual of Mental Disorders, Fourth Edition, which changed many of the diagnostic criteria for Major Depressive Disorder. Items involving changes in body image, hypochondria, and difficulty working were replaced. Also, sleep loss and appetite loss items were revised to assess both increases and decreases in sleep and appetite. All but three of the items were reworded; only the items dealing with feelings of being punished, thoughts about suicide, and interest in sex remained the same. Finally, participants were asked to rate how they had been feeling for the preceding 2 weeks. The BDI-II contains 21 questions, each answer being scored on a scale from 0 to 3. When the test is scored, a value of 0 to 3 is assigned for each answer and the total score is compared to a key to determine the level of depression. The standard cut-offs are as follows: 0–13: minimal depression; 14–19: mild depression; 20–28: moderate depression; and 29–63: severe depression. Higher total scores indicate more severe depressive symptoms [13,14]. A previous study reported that the BDI-II is positively correlated with the Hamilton Depression Rating Scale with a Pearson correlation coefficient of 0.71, showing good agreement, and the test also has a high 1-week test–retest reliability (Pearson r = 0.93), suggesting that it is not overly sensitive to daily variations in mood [15]. The test also has high internal consistency (α = 0.91) [14].

#### 2.2.5. Short-Form 36-Item Health Survey

The Short-Form 36 (SF-36) is a generic measure because it assesses health concepts that represent basic human values relevant to everyone’s functional status and well-being [16]. A Chinese Taiwanese version of the SF-36 was used in our study [17,18,19]. This assessment is extensively documented in reports from studies of clinical patients and general populations as reliable and valid [16,20,21,22]. The SF-36 comprises 36 questions covering eight aspects of health status: physical functioning (PF), role-physical (role limitations due to physical health problems; RP), bodily pain (BP), general health (GH), vitality (VT), social functioning (SF), role-emotional (role limitations due to emotional problems; RE), and mental health (MH) [16,20,21]. The scores of questions relating to each scale are summed and rescaled to a 100-point scale, where 100 is the best possible score and 0 the worst possible score. The eight scales in the SF-36 are further divided into a two-component summary: a Physical Component Summary (PCS) and a Mental Component Summary (MCS) [22]. The PCS and MCS are standardized so that the mean is set to 50 and one standard deviation is equal to 10 points. The details of the eight scales and two summary components have been systematically documented elsewhere [21,22]. In this study, internal consistency of the SF-36 subscale scores was good with Cronbach’s alpha exceeding 0.80 for each scale, except social function scale (Cronbach’s alpha = 0.78) and mental health scale (Cronbach’s alpha = 0.77).

### 2.3. Dependent and Independent Variables

The primary independent variables of interest were perceived work-related stress, medical history, self-reported physical and mental conditions (depressive symptoms and sleep status), and personal lifestyle behavior (smoke, alcohol, and exercise). The dependent variables in this study were PCS and MCS scores. For the PCS, very low scores indicate substantial limitations in self-care, physical, social, and role activities; severe body pain; or frequent tiredness [22]. For the MCS, very low scores indicate frequent psychologic distress, and substantial social and role disability due to emotional problems [22]. 

### 2.4. Statistical Analysis

All analyses were performed using Statistical Analysis System (SAS 6.12; SAS Institute, Cary, NC, USA) software. Participants’ profiles among white-collar migrant workers were reported. Analyses were based on a published scoring algorithm for the BDI-II and SF-36 accepted guidelines [13,14,21,22]. Bivariate analyses were performed between the perceived work-related stress and other independent variables using the chi-square test. The Shapiro-Wilk test of normality revealed that all of the scales of the SF-36 among our sample were negatively skewed and showed a positive kurtosis distribution (*P* < 0.01). Profiles of the 8 scales of the SF-36 among subjects with perceived work-related stress, alcohol consumption, self-reported neck pain, poor sleep, and depression among white-collar Taiwanese workers in China were thus evaluated using the Mann-Whitney test. A *P *value of less than 0.05 was considered statistically significant. 

To determine different risk factors, including lifestyle and work-related stress, medical history, and self-reported symptoms for HRQoL among white-collar migrant workers, a multivariate analysis was used to identify subject characteristics that were independently associated with the summary scores (PCS and MCS) of the SF-36 using separate multiple linear regression models after adjusting for demographic variables. Six sets of regression models were separately calculated controlling for demographic variables. The first to third models estimated the relationship between lifestyle and perceived work-related stress, medical history and sleep problems, and self-report symptoms and PCS, respectively. Models four to six estimated the relationship between lifestyle and perceived work-related stress, medical history and sleep problems, and self-report symptoms and MCS, respectively. We chose six models because the results would more clearly render which risk factor is more important among similar types of predictors and the risk factors that warranted immediate attention.

## 3. Results

The descriptive characteristics of participants in this study are shown in Table 1. The participating subjects were more likely to be men (78.9%) and young (≤40 years old: 54.3%), with a high education level (84.6%), and married and living with their spouse (50.0%). The prevalence of current smoking and alcohol habits was 31.4% and 72.4%, respectively. The prevalence of regular exercise was 12.2% and the percentage of subjects with perceived work-related stress was 62.2%. Health status among participants included hyperlipidemia in 16%, gastric ulcer in 12.8%; self-reported symptoms of neck pain in 16.7% and low back pain in 18.0%; poor sleep in 19.2%; and with moderate or higher level of depression in 7.7%. 

**Table 1:** Descriptive characteristics of participants in this study among white-collar Taiwanese workers in China (n = 156).

Variables			n	%
**Demographics**				
	Sex	Male	123	78.9
		Female	33	21.1
	Age (years)	≤40	82	54.3
		41-50	53	35.1
		>50	21	10.6
	Education	High education and below	24	15.4
		College and above	132	84.6
	Marital status	Single	32	20.5
		Married and living with their spouse	78	50.0
		Married and living without their spouse	31	19.9
		divorced/separated	15	9.6
	Monthly income ($)	1,600-2,000	67	44.0
		2,001-2,400	32	20.5
		Above 2,401	57	36.5
**Lifestyle and work-related stress**				
	Smoking status (%)	Current smoking	49	31.4
	Alcohol	Yes	113	72.4
	Regular exercise	Yes	19	12.2
	Perceived work-related stress	Yes	97	62.2
**Medical history**				
	Diabetes	Yes	3	2.0
	Hypertension	Yes	14	8.9
	Cardiovascular disease	Yes	4	2.6
	Hyperlipidemia	Yes	25	16.0
	Metabolic arthritis	Yes	14	9.0
	Gastric ulcer	Yes	20	12.8
**Self-reported symptoms**				
Neck pain		Yes	26	16.7
Low back pain		Yes	28	18.0
	Headache	Yes	17	10.9
	Poor sleep	Yes	30	19.2
	BDI scores	0-13: minimal depression	130	83.3
		14-19: mild depression	14	9.0
		20-63: moderate/severe depression	12	7.7

A comparison of the demographic information and some of the variables in the subjects with and without perceived work-related stress is shown in Table 2. The findings revealed that subjects with greater levels of perceived work-related stress consumed more alcohol than those who did not perceive work-related stress (78.4% *vs.* 63.8%, *P* = 0.048); they also reported more hyperlipidemia (*P* = 0.045), greater neck pain (*P* = 0.009), more poor sleep (*P* = 0.0315), and more mild /moderate/severe depression (*P* = 0.009) than those who did not perceive work-related stress. There were no significant differences between the groups among the other variables. 

**Table 2:** Association between alcohol, hyperlipidemia, self-reported neck pain, poor sleep status, depression, and perceived work-related stress among white-collar Taiwanese workers in China, 2007 (n = 156).

Variables	Perceived Work-Related Stress %	Non-Perceived Work-Related Stress %	*P*-Value
Alcohol			
Yes	78.4	63.8	0.048
No	21.6	36.2	
Hyperlipidemia			
Yes	20.6	8.5	0.045
No	79.4	91.5	
Self-reported neck pain			
Yes	22.7	6.8	0.009
No	77.3	93.2	
Poor sleep			
Yes	24.7	10.5	0.0315
No	75.3	89.5	
Depression (BDI scores)			
Minimal depression	77.3	93.2	0.009
Mild/moderate/severe depression	22.6	6.8	

Other independent variables in this study (demographics, medical history, lifestyle, and self-reported symptoms) were not significantly associated with perceived work-related stress.

**Figure 1:**
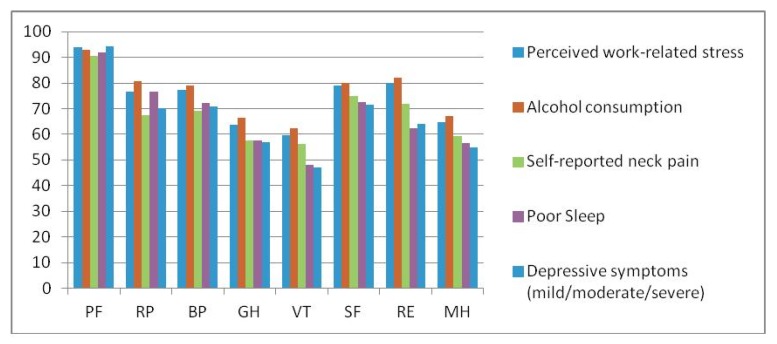
Profiles of the 8 SF-36 scales among subjects reporting perceived work-related stress, alcohol consumption, self-reported neck pain, poor sleep, and depressive symptoms among white-collar Taiwanese workers in China.

The white-collar migrant workers’ 8 scales of the SF-36 according to different health conditions are shown in Table 3. Figure 1 shows the profiles of the 8 SF-36 scales among subjects with perceived work-related stress, or who consumed alcohol, reported neck pain, poor sleep, and depression among white-collar Taiwanese workers in China. Table 4 presents the results from a series of multiple regression analysis models evaluating independent predictors of the PCS and MCS from the SF-36 adjusting for demographic variables. To determine lifestyle and work-related stress variables and HRQoL, perceived work-related stress negatively impacted PCS (β = −2.68; *P* = 0.010) and MCS (β = −3.92; *P* = 0.022) scores. Subjects with hyperlipidemia had significantly lower PCS scores (β = −3.79; *P* = 0.011), whereas cardiovascular disease, gastric ulcer, and poor sleep were associated with statistically lower MCS scores (*P* < 0.05). Migrant workers that reported neck pain had significantly lower PCS scores (β = −5.38; *P* < 0.0001). Having a depressive condition was associated with more negative PCS and MCS scores.

**Table 3:** Comparison of health status of the SF-36 among white-collar Taiwanese workers in China.

	SF-36 (scores)							
	Physical Function	Role Limitations: Physical	Bodily Pain	General Health Perceptions	Vitality/Energy	Social Function	Role Limitations: Emotional	Mental Health
**All participants**	92.7 ± 13.6	80.8 ± 34.3	81.1 ± 17.5	67.2 ± 17.1	62.4 ± 17.9	80.5 ± 14.4	82.6 ± 33.7	66.7 ± 15.0
Perceived work-related								
stress								
Yes	93.8 ± 9.7	76.5 ± 36.5 *	77.4 ± 17.6 *	63.7 ± 17.2 *	59.5 ± 18.4 *	78.9 ± 14.2	80.0 ± 35.2	64.8 ± 14.3
No	90.8 ± 18.3	88.1 ± 29.1	87.3 ± 15.7	72.6 ± 15.5	67.2 ± 16.2	83.1 ± 14.6	87.1 ± 30.7	69.8 ± 15.9
Alcohol consumption								
Yes	92.9 ± 13.9	80.6 ± 33.6	79.0 ± 17.3 *	66.5 ± 17.2	62.2 ± 18.4	79.9 ± 14.3	81.9 ± 33.8	67.2 ± 14.4
No	91.8 ± 12.9	80.9 ± 36.9	86.6 ± 17.3	68.3 ± 16.0	62.5 ± 16.4	81.5 ± 14.7	84.1 ± 33.9	65.2 ± 16.7
Self-reported neck pain								
Yes								
No	90.7 ± 12.6	67.3 ± 39.8 *	69.1 ± 12.6 *	57.6 ± 16.2 *	56.2 ± 14.6 *	75.0 ± 14.8	71.7 ± 39.6	59.3 ± 12.9 *
	93.1 ± 13.8	83.5 ± 32.6	83.6 ± 17.4	69.3 ± 16.6	63.7 ± 18.3	81.6 ± 14.2	84.8 ± 32.0	68.3 ± 15.0
Poor sleep								
Yes	92.0 ± 11.2	76.6 ± 35.9	72.2 ± 18.6 *	57.6 ± 15.0 *	48.1 ± 15.2 *	72.7 ± 15.9 *	62.2 ± 45.2 *	56.5 ± 14.6 *
No	92.8 ± 14.2	81.7 ± 34.1	83.3 ± 16.7	68.7 ± 16.7	65.4 ± 16.9	82.1 ± 13.5	87.5 ± 28.4	69.0 ± 14.1
Depression								
Yes	94.2 ± 68.8	70.1 ± 40.6	70.9 ± 15.5 *	57.0 ± 9.5 *	46.9 ± 13.5 *	71.6 ± 16.4 *	64.1 ± 43.1 *	54.7 ± 10.1 *
No	92.4 ± 14.6	83.0 ± 32.7	83.1 ± 17.3	69.4 ± 17.5	65.7 ± 17.0	82.4 ± 13.3	86.4 ± 30.2	69.1 ± 14.7

* Statistically significant difference (p < 0.05) by using Mann-Whitney Test.

**Table 4:** Regression coefficients of SF-36 scales with work pressure and health status among white-collar Taiwanese workers in China, 2007.

		PCS ß (*p*-value)			MCS ß (*p*-value)		
		Model 1	Model 2	Model 3	Model 4	Model 5	Model 6
**Lifestyle and perceived work-related stress ** *							
	Smoking (current smoking)	N.S.			N.S.		
	Alcohol consumption (yes)	N.S.			N.S.		
	Regular exercise (yes)	N.S.			N.S.		
	Perceived work-related stress (yes)	−2.68 (*p *= 0.010)			−3.92 (*p *= 0.022)		
**Medical history and sleep problems ** *							
	Diabetes (yes)		N.S.			N.S.	
Hypertension (yes)			N.S.			N.S.	
Cardiovascular disease (yes)			N.S.			−13.6 (*p *= 0.018)	
	Hyperlipidemia (yes)		−3.79 (*p *= 0.011)			N.S.	
Metabolic arthritis (yes)			N.S.			N.S.	
Gastric ulcer (yes)			N.S.			−6.09 (*p *= 0.014)	
	Poor sleep (yes)		N.S.			−5.55 (*p *= 0.013)	
**Self-report symptoms ** *							
	Neck pain (yes)			−5.38 (*p* < 0.0001)			N.S.
Low back pain (yes)				N.S.			N.S.
Headache (yes)				N.S.			N.S.
Depression (mild/moderate/severe)				−1.92 (*p* = 0.114)			−8.57 (*p *< 0.0001)

* Adjusted demographics variables included sex, age, education, and income. N.S.: non-significant.

## 4. Discussion

The majority of previous studies evaluated blue-collar migrant workers’ health because they often serve as the labor force that fills the “3 D” jobs (dangerous, dirty, and degrading). At the same time, globalization of the economy and competition of the international market has led to an increase in migration, particularly for white-collar workers. The overall impact of immigration on white-collar workers’ health is poorly understood. In this study, we focused on white-collar workers and found: (1) a high prevalence of alcohol consumption (72.4%) and perceived work-related stress (62.2%) and a lower prevalence of regular exercise (12.2%). People’s susceptibility to disease varies widely and may be a reflection of differences in their biologic predisposition, personality, behavior, and environmental exposure. Based on these data, unhealthy determinant factors were indeed detected in our sample of workers and are dangerous to their health, e.g., continuing work-related stress, greater alcohol intake (regardless of reasons such as social intercourse for business or personal behavior), and lower levels of physical activities that reduce both heart and lung functions all had negative impact on their health and decreased HRQoL. (2) We found that subjects with perceived work-related stress that consumed more alcohol and those with hyperlipidemia had higher incidence of self-reported neck pain, poor sleep, and mild/moderate/severe depression. Multivariate analysis revealed a significant association between work-related stress and neck pain (*P* = 0.0302) and depression (*P* = 0.0485; data not shown). Similar findings from Japan [23] indicated that worker job stress indices are highly correlated with tension-anxiety and depression, and workers who are under higher job demand and strain are less likely to exhibit healthy behaviors, possibly due to emotional strain. (3) The present study found that, except for higher scores on the scale of physical health, subjects scored lower than expected on the scale of general heath perceptions, vitality/energy, and mental health; and perceived work-related stress and a feeling of depression were related to lower PCS and MCS scores of the SF-36 after adjusting for other demographic variables. These results demonstrated a possibly negative impact on the quality of life among the white-collar migrant workers. Psychological stress is commonly believed to play an important role in illness and premature death [24]. Hence, the industry occupational health staff should concern about the white-collar workers’ outcome of unhealthy lifestyle and behavior and work-related stress under international competition. To prevent impaired HRQoL and premature death, white-collar migrant workers, particularly white-collar Asian migrant workers, must pay more attention to work-related stress issues in their working environment because workers often ignore and do not seek professional consulting services. 

According to the World Health Organization, there is a close link between the level of safety and health, socioeconomic development and quality of life, and well-being of working people. As a consequence, reducing exposure to adverse conditions at work is likely to have a positive and productive impact on the physical and mental HRQoL of the working population [25]. For this reason, HRQoL has also been used as an outcome in studies of work [25,26,27,28]. A study of the HRQoL of financial service employees in Brazil found that overcommitment was associated with poor HRQoL in the physical and mental domain [25]. Automotive assembly workers in Malaysia [27] reported that job stress is directly related to anxiety and depression, and inversely related to physical health, environment conditions, and social relationships in the World Health Organization Quality of Life-Brief (WHOQOL-BREF) study. The Poland study [28] further revealed that psychosocial conditions of work significantly influence HRQoL at the beginning of older age. As noted earlier, from the perspective of preventive medicine, identification of workers with early stages of perceived job stress or unhealthy behavior and those who are at risk for developing adverse physical and mental conditions may provide an opportunity to prevent the deterioration of HRQoL. The present study also suggested an association between perceived work-related stress and unhealthy behavior, particularly with regard to alcohol intake among white-collar migrant workers. The work environment is probably an important source of stress for most adults. This means that lifestyle modification is no longer an individual issue but within the purview of occupational health professionals. Various psychologic interventions such as stress management may be helpful in decreasing the level of perceived work-related stress. 

Our data revealed a significant association between work-related stress and neck pain (*P* = 0.0302, data not shown), while neck pain negatively impacted PCS scores of the SF-36 (β= −5.38; *P *< 0.0001). Previous reports also demonstrated strong relationships between psychologic distress, job demands (stressful work, hectic work), low job control, and pain at multiple sites [29]. Although neck pain is not fatal, it greatly influences daily social or work activities and the quality of life of the worker [30,31,32]. Individual work styles in response to workload demands and stressors, including working with heightened muscle tension and mental fatigue, were significantly associated with musculoskeletal symptoms [30]. Similar results were reported in the 2008 US National Health and Wellness Survey [31]: arthritis, back pain, and fibromyalgia pain were all associated with significantly lower levels of HRQoL, and all pain conditions were associated with a greater loss of work, even after adjusting for demographic and health characteristics. Another American productivity survey revealed that common pain conditions appear to have an adverse effect on work, and lost productive time from common pain conditions among active workers costs an estimated 61.2 billion US dollars per year [32]. Thus, these results support a role for job stress as a risk factor for musculoskeletal symptoms and impaired work-related HRQoL. In addition, the dispositional characteristics of the individual, in combination with the work environment, are importantly related to disease progression and impaired HRQoL. 

## 5. Conclusions

In the present study, white-collar employee health was used as the starting point to conduct a preliminary survey. We used a relatively homogenous sample of subjects who were similar in ethnicity and sociodemographic characteristics. On the other hand, there are some limitations to our study. First, this study was cross-sectional in design; therefore, only association and not causation could be evaluated. Second, in the beginning, this study focused on exploring white-collar migrant workers’ HRQoL and its related risk factors. We underestimated the impact of job stress on HRQoL among white-collar migrant workers, however, and therefore neglected to use a standardized instrument for measuring job stress. The dichotomous variable that we used is not likely to provide nearly enough information. For the assessment of socioeconomic status, co-morbidities and work-related stress, we used a dichotomized classification, which was overly simplistic and might have biased the results. Future studies should adopt a richer “work-related stress” instrument to measure these factors to obtain more detailed data, which would be much more valuable. 

Third, a selection bias due to healthy-worker effect was inevitable. Excluding those who refused to participate and could not be reached possibly due to a health issue or medical leave, removes a disproportionate number of workers with declining HRQoL, and may have biased the results of the study. This would lead to an underestimation of the quality of life scores and reduce the strength of the associations. In other words, excluded subjects likely included more unhealthy subjects than included subjects; hence, the impact on HRQoL is likely to be greater than our results indicate. 

The research indicates that white-collar migrant workers are young with a high socioeconomic status compared with blue-collar migrant workers. In the present study, however, we found that except for higher physical health scores, the subjects scored lower than expected on scales of general heath perceptions, vitality/energy, and mental health. Subjects with perceived work-related consumed more alcohol and more frequently reported a history of hyperlipidemia, with more self-reports of neck pain, poor sleep, and mild/moderate/severe depression. A significant association between perceived work-related stress and depressive status indirectly affects HRQoL. 
